# Exploring the Link Between Early Technology Exposure and Developmental Milestones in Childhood

**DOI:** 10.7759/cureus.71791

**Published:** 2024-10-18

**Authors:** Justin B Atkins, Samantha Difulvio, Jordana Boneh, Rebecca Myers, Caroline Tohic, Crystal Dickson, Diana Pena, Edward Simanton

**Affiliations:** 1 College of Medicine, Kirk Kerkorian School of Medicine at University of Nevada, Las Vegas (UNLV), Las Vegas, USA; 2 Pediatrics, Kirk Kerkorian School of Medicine at University of Nevada, Las Vegas (UNLV), Las Vegas, USA; 3 Diagnostic Radiology, Oregon Health and Science University School of Medicine, Portland, USA; 4 Pediatrics, University of California San Francisco, San Francisco, USA; 5 Pediatrics, Stanford University, Stanford, USA; 6 Medical Education, Kirk Kerkorian School of Medicine at University of Nevada, Las Vegas (UNLV), Las Vegas, USA

**Keywords:** child behaviour and development, child development, child's screen time, pediatric milestones, screen-based simulation, screen exposure time, screen time

## Abstract

Background

This study examined the relationship between touchscreen exposure and developmental outcomes in early childhood using the Ages and Stages Questionnaire, Third Edition (ASQ-3), a widely-used developmental screening tool.

Methods

During a well-child visit, parents of 51 children aged 18-36 months completed the ASQ-3 and a survey on their child's touchscreen habits (timing of introduction, daily usage duration, independent use, engagement in creative activities). Medical records were reviewed for developmental diagnoses and health conditions.

Analysis

One-way analysis of variances (ANOVAs) examined differences in ASQ-3 domain scores (problem-solving, personal-social, communication, gross motor, fine motor) based on the age of touchscreen introduction and average daily usage time. Independent t-tests compared scores between groups defined by independent touchscreen use and creative activity engagement. Effect sizes were calculated for significant differences.

Results

In the problem-solving domain, children introduced to touchscreens before 12 months of age scored significantly higher than those introduced later, with a statistical significance of p<0.05 and an effect size of d=0.45. However, there were no significant differences in scores based on the duration of daily touchscreen usage. In the personal-social domain, children introduced to touchscreens at 12 months or later scored significantly higher than those introduced before 12 months, with a significance of p<0.05 and an effect size of d=0.51. Moderate daily usage of 30-90 minutes was associated with higher scores compared to lower usage of less than 30 minutes or higher usage of more than 90 minutes (p<0.05). Additionally, children who used touchscreens independently had significantly higher scores, with a significance of p<0.01 and an effect size of d=0.76. Those who engaged in activities such as coloring or crafting also had significantly higher scores, with a significance of p<0.01 and an effect size of d=0.82. In other domains, such as communication, gross motor, and fine motor, no significant effects of touchscreen exposure were observed.

Discussion

Earlier touchscreen introduction may facilitate problem-solving skills like experimentation and cognitive flexibility through interactive digital play. However, excessive touchscreen use (greater than 90 minutes per day) before 12 months could interfere with pivotal social-emotional learning from real-world interactions, impeding personal-social development. Moderate, supervised touchscreen exposure combined with creative hands-on activities appears optimal for fostering strong personal-social competencies like cooperation and emotional understanding during the toddler years.

Conclusion

Timing, duration, context, and content of touchscreen experiences in early childhood relate differently to specific developmental domains. Guidelines should consider these nuances to support overall healthy child development in our digital world. Larger longitudinal studies using multi-method assessments are needed.

## Introduction

The integration of touchscreen devices into our everyday lives has become a part of life that aids in our learning, social connectedness, and leisure time activities [1]. The use of touchscreens and time spent viewing electronic devices is a contentious topic regarding the pediatric population [2]. Touchscreen devices are believed by some to assist in the development of fine motor skills, a finding which was supported by research for developmental milestones between six and 36 months [3]. Electronic devices are touted as a way for children to engage with educational content. A quick search on a device’s application distribution platform will identify the best education apps for children. It may also be easier for young children to engage more effectively with a touchscreen than with computers and laptops, which require more refined motor skills. As such, finding ways to incorporate the use of this technology into the lives of children has been recommended by extensive research into this topic. Parental guidance and shared viewing of touchscreen devices can be an effective strategy to yield positive outcomes for pediatric populations in regard to touchscreen use [4-6].

However, other studies have found a negative association between increasing screen time and the achievement of developmental milestones [6,7]. A common critique of critics is that touchscreens may not be effective if they reduce the time children spend engaging with adults and other children face-to-face [8]. Several recommendations have been made as to how not to implement this technology into the life of a child [8].

Despite a lack of clear evidence as to the effects of screen time on young children, medical organizations are stepping in to make recommendations for parents. Both the World Health Organization and the American Academy of Pediatrics have released guidelines limiting screen time in the youngest children and encouraging discourse about screen time between parents and older children [9].

Incorporating the use of touchscreen devices while adhering to recommendations and limitations can be useful for pediatric populations [10]. The issue with this is that several studies have found that recommendations are not generally adhered to [11].

## Materials and methods

Participants

The study included 51 children aged 23-25 months (mean age = 24.2 months, standard deviation (SD) = 0.8 months) who attended a well-child visit at the Department of Pediatrics, University of Nevada, Las Vegas (UNLV) School of Medicine between November 2020 and May 2021. Children were excluded if they had diagnosed developmental delays, hearing/vision impairments, autism spectrum disorder, attention-deficit/hyperactivity disorder, or other chronic conditions that could impact developmental screening results. Parents/legal guardians provided written informed consent for their child's participation as well as authorization to review the child's medical record.

Measures

Parents completed the 24-month Ages and Stages Questionnaire, Third Edition (ASQ-3), a validated developmental screening tool assessing skills across five domains: communication, gross motor, fine motor, problem-solving, and personal-social [12]. The ASQ-3 requires parents to report whether their child has acquired specific developmental milestones (yes/sometimes/not yet) [12]. Higher scores indicate more advanced development.

A supplemental survey collected data on child demographics (age, gender, race/ethnicity); primary language spoken at home; caregiver education level; child's school/daycare attendance; number of touchscreen devices in the home; age when the child was first introduced to touchscreens; average daily touchscreen usage time; whether child usually used touchscreens alone; engagement in coloring/crafting/art activities. Parental education level was also included to assess for socioeconomic factors. Medical records were then reviewed for the presence of developmental diagnoses and chronic health conditions.

Procedure

During the well-child visit, parents independently completed the paper ASQ-3 questionnaire and supplemental survey on touchscreen exposure/usage while in the waiting room. A trained research assistant was available to clarify any questions. Parents were instructed to try each ASQ-3 activity with their child before responding.

ASQ-3 responses were scored per standardized guidelines, with "yes" = 10 points, "sometimes" = 5 points, and "not yet" = 0 points. Item scores were summed to calculate total scores for each developmental domain, with higher scores indicating more advanced skills.

Analysis

Descriptive statistics were used to summarize the sample characteristics and ASQ-3 domain scores. One-way analysis of variances (ANOVAs) was conducted to examine differences in ASQ-3 scores based on the age of touchscreen introduction, categorized as before 12 months, at 12 months, and after 12 months, as well as average daily usage time, categorized as less than 30 minutes, 30 to 90 minutes, and more than 90 minutes. Independent samples t-tests were utilized to compare ASQ-3 scores between groups defined by whether the child usually used touchscreens alone (yes/no) and whether the child engaged in activities such as coloring, crafting, or art (yes/no).

Levene's test was employed to assess the equality of variances before conducting the t-tests. Effect sizes, measured by Cohen's d, were calculated for any significant group differences. An alpha level of 0.05 was used to determine statistical significance for all analyses, which were performed using the Statistical Package for the Social Sciences (IBM SPSS Statistics for Windows, IBM Corp., Version 27, Armonk, NY). Missing data were addressed through pairwise deletion.

## Results

The results in Table 1 show the mean scores and SDs for the ASQ problem-solving and personal-social domains, broken down by when children were introduced to touchscreens.

**Table 1:** ASQ problem-solving and personal-social domains ASQ: Ages and Stages Questionnaire

		N	Mean	Std. Deviation	Std. Error
						ASQ problem-solving score	Does not use touch screens	9	35.0000	20.00000	6.66667
Introduced before age 12 months	27	55.5556	9.12871	1.75682		
Introduced at 12 months or later	15	51.6667	10.29332	2.65772		
Total	51	50.7843	13.94176	1.95224		
ASQ personal-social score	Does not use touch screens	9	35.0000	11.72604	3.90868
	Introduced before age 12 months	27	52.5926	8.36319	1.6095
	Introduced at 12 months or later	14	54.6429	7.71220	2.06117
	Total	50	50.0000	11.24858	1.59079

In the ASQ problem-solving domain, children who did not use touchscreens had a mean score of 35.0 with an SD of 20.0. In contrast, children introduced to touchscreens before 12 months had the highest mean score of 55.56 with an SD of 9.13, while those introduced at 12 months or later had a mean score of 51.67 with an SD of 10.29.

For the ASQ personal-social domain, children who did not use touchscreens again had the lowest mean score of 35.0, with an SD of 11.73. Children introduced to touchscreens before 12 months had a mean score of 52.59 with an SD of 8.36, whereas those introduced at 12 months or later had the highest mean score of 54.64 with an SD of 7.71.

These results suggest that introducing touchscreens before 12 months may be associated with higher problem-solving scores, while introducing them at 12 months or later may be linked to higher personal-social scores on the ASQ screening tool. The one-way ANOVA analysis presented in Table 2 details these findings based on the timing of the touchscreen introduction.

**Table 2:** One-way ANOVA analysis for the ASQ problem-solving and personal-social scores based on when children were introduced to touchscreens ANOVA: analysis of variance; ASQ: Ages and Stages Questionnaire

		Sum of Squares	df	Mean Square	F	Sig.
ASQ problem-solving score	Between Groups	2868.627	2	1434.314	10.051	<0.001
	Within Groups	6850.000	48	142.708	-	-
	Total	9718.627	50	-	-	-
ASQ personal-social score	Between Groups	2508.267	2	1254.134	15.967	<0.001
	Within Groups	3691.733	47	78.548	-	-
	Total	6200.000	49	-	-	-

The analysis of the ASQ problem-solving scores revealed a between-groups sum of squares of 2868.627 with 2 degrees of freedom and a within-groups (error) sum of squares of 6850.000 with 48 degrees of freedom. The resulting F-statistic is 10.051, which is statistically significant (p < 0.001). This indicates a statistically significant difference in the ASQ problem-solving scores between the three groups based on the age of touchscreen introduction.

Similarly, for the ASQ personal-social scores, the between-groups sum of squares is 2508.267 with 2 degrees of freedom, and the within-groups (error) sum of squares is 3691.733 with 47 degrees of freedom. The F-statistic for this domain is 15.967, also statistically significant (p < 0.001). This signifies a statistically significant difference in the ASQ personal-social scores between the three groups based on when touchscreens were introduced.

The significant F-statistics for both ASQ domains suggest that the mean scores differ based on the timing of touchscreen introduction, with some groups scoring higher than others in problem-solving and personal-social skills. The results presented in Table 3 provide the descriptive statistics for the ASQ problem-solving and personal-social scores, categorized by the amount of time children spent using touchscreens per day.

**Table 3:** Descriptive statistics for the ASQ problem-solving and personal-social scores ASQ: Ages and Stages Questionnaire

		N	Mean	Std. Deviation	Std. Error	Maximum
ASQ problem-solving score	0 to less than 30 minutes	17	47.0588	17.23539	4.18020	60
	30 to 90 minutes	23	55.2174	10.60427	2.21114	90
	more than 90 minutes	11	47.2727	12.91581	3.89426	60
	Total	51	50.7843	13.94176	1.95224	90
ASQ personal-social score	0 to less than 30 minutes	17	44.1176	12.89807	3.12824	60
	30 to 90 minutes	22	53.4091	7.46203	1.59091	60
	more than 90 minutes	11	52.2727	12.11686	3.65337	60
	Total	50	50.0000	11.24858	1.59079	60

In the ASQ problem-solving domain, children who used touchscreens for 0 to less than 30 minutes per day had a mean score of 47.06 with an SD of 17.24. Those who used touchscreens for 30 to 90 minutes per day achieved the highest mean score of 55.22 with an SD of 10.60. Meanwhile, children using touchscreens for more than 90 minutes per day had a mean score of 47.27 with an SD of 12.92. The maximum possible score for the problem-solving domain was 90.0.

In the ASQ personal-social domain, children who used touchscreens for 0 to less than 30 minutes per day had the lowest mean score of 44.12 with an SD of 12.90. Children using touchscreens for 30 to 90 minutes per day had a mean score of 53.41 with an SD of 7.46, while those using them for more than 90 minutes per day had a mean score of 52.27 with an SD of 12.12. The maximum score for the personal-social domain was 60.0.

These results suggest that moderate touchscreen use (30 to 90 minutes per day) may be associated with higher scores in both the problem-solving and personal-social domains of the ASQ screening tool compared to low or high touchscreen use. The results in Table 4 present the one-way ANOVA analysis for the ASQ problem-solving and personal-social scores based on the amount of time children spent using touchscreens per day.

**Table 4:** One-way ANOVA analysis for the ASQ problem-solving and personal-social scores based on the amount of time children spent using touchscreens per day ANOVA: analysis of variance; ASQ: Ages and Stages Questionnaire

		Sum of Squares	df	Mean Square	F	Sig.
ASQ problem-solving score	Between Groups	823.591	2	411.796	2.222	0.119
	Within Groups	8895.036	48	185.313	-	-
	Total	9718.627	50	-	-	-
ASQ personal-social score	Between Groups	900.735	2	450.368	3.994	0.025
	Within Groups	5299.265	47	112.750	-	-
	Total	6200.000	49	-	-	-

In the ASQ problem-solving domain, the between-groups sum of squares is 823.591 with 2 degrees of freedom, while the within-groups (error) sum of squares is 8895.036 with 48 degrees of freedom. The resulting F-statistic is 2.222, which is not statistically significant (p = 0.119). This indicates that there is no statistically significant difference in the ASQ problem-solving scores between the three groups based on touchscreen usage time.

In contrast, the ASQ personal-social domain shows a between-groups sum of squares of 900.735 with 2 degrees of freedom and a within-groups (error) sum of squares of 5299.265 with 47 degrees of freedom. The F-statistic for this domain is 3.994, which is statistically significant (p = 0.025). This suggests a statistically significant difference in the ASQ personal-social scores between the three groups based on touchscreen usage time.

While the groups did not differ significantly in problem-solving scores, there were significant differences in personal-social scores depending on how much time children spent using touchscreens per day. The results in Table 5 show the group statistics for the ASQ problem-solving and personal-social scores, broken down by whether children usually used touchscreens alone or not.

**Table 5:** Group statistics ASQ: Ages and Stages Questionnaire

	Usually Uses Touchscreen Alone	N	Mean	Std. Deviation	Std. Error Mean
ASQ problem-solving score	Yes	13	52.3077	9.707250	2.692310
	No	38	50.2632	15.199010	2.465610
ASQ personal-social score	Yes	13	57.3077	4.837090	1.341570
	No	37	47.4324	11.762800	1.933790

In the ASQ problem-solving domain, children who usually used touchscreens alone had a mean score of 52.31 with an SD of 9.71. In comparison, children who did not usually use touchscreens alone had a slightly lower mean score of 50.26 with an SD of 15.20.

In the ASQ personal-social domain, children who usually used touchscreens alone had a higher mean score of 57.31 with an SD of 4.84. Conversely, children who did not usually use touchscreens alone had a lower mean score of 47.43 with an SD of 11.76.

These results suggest that children who usually used touchscreens alone tended to score higher on both the problem-solving and personal-social domains of the ASQ screening tool compared to those who did not. The difference in mean scores was more pronounced for the personal-social domain. The results in Table 6 present the outcomes of Levene's test for equality of variances and the independent samples t-test for the ASQ problem-solving and personal-social scores.

**Table 6:** Outcomes of Levene's test for equality of variances and the independent samples t-test for the ASQ problem-solving and personal-social scores ASQ: Ages and Stages Questionnaire

		Levene's Test for Equality of Variances		t-test for Equality of Means					
		F	Sig.	t	df	Significance		Mean Difference	Std. Error Difference
						One-Sided p	Two-Sided p		
ASQ problem-solving score	Equal variances assumed	0.68	0.414	0.453	49.000	0.326	0.653	2.04453	4.51564
	Equal variances not assumed	-	-	0.560	33.033	0.290	0.579	2.04453	3.65072
ASQ personal-social score	Equal variances assumed	9.101	0.004	2.925	48.000	0.003	0.005	9.87526	3.37568
	Equal variances not assumed	-	-	4.196	46.605	<0.001	<0.001	9.87526	2.35358

Levene's test for equality of variances assesses whether the variances of two groups being compared are equal. For the ASQ problem-solving score, the test yielded an F-value of 0.680 with a p-value of 0.414. Since the p-value is greater than 0.05, we fail to reject the null hypothesis, indicating that the variances are equal. In contrast, for the ASQ personal-social score, the test resulted in an F-value of 9.101 with a p-value of 0.004. Since the p-value is less than 0.05, we reject the null hypothesis and conclude that the variances are unequal.

The independent samples t-test evaluates whether the mean scores of the two groups differ significantly. For the ASQ problem-solving score, assuming equal variances, the t-test yielded t(49) = 0.453 with a p-value of 0.653. When not assuming equal variances, the result was t(33.033) = 0.560 with a p-value of 0.290. In both cases, since the p-values are greater than 0.05, the mean scores do not differ significantly between groups. For the ASQ personal-social score, assuming equal variances, the t-test showed t(48) = 2.925 with a p-value of 0.005. Without assuming equal variances, the result was t(46.605) = 4.196 with a p-value of less than 0.001. Since Levene's test indicated unequal variances, we refer to the "Equal variances not assumed" row. The mean difference is 9.88 with a p-value of less than 0.001, indicating a statistically significant difference in personal-social scores between groups.

In summary, while the groups did not differ on problem-solving scores, there was a significant difference in personal-social scores, with the assumption of equal variances being violated for that domain. The results in Table 7 show the group statistics for the ASQ problem-solving and personal-social scores, broken down by whether children engaged in coloring, crafting, or art activities.

**Table 7:** Group statistics for the ASQ problem-solving and personal-social scores, broken down by whether children engaged in coloring, crafting, or art activities ASQ: Ages and Stages Questionnaire

	Coloring, Crafting, or Art Activities	N	Mean	Std. Deviation	Std. Error Mean
ASQ problem-solving score	Yes	21	53.5714	12.26202	2.67579
	No	30	48.8333	14.89523	2.71948
ASQ personal-social score	Yes	20	55.7500	6.34014	1.41770
	No	30	46.1667	12.22514	2.23199

The results present the group statistics for the ASQ problem-solving and personal-social scores, categorized by whether children engaged in coloring, crafting, or art activities.

For the ASQ problem-solving score, children who participated in these activities had a higher mean score of 53.57 with an SD of 12.26. In contrast, children who did not engage in such activities had a lower mean score of 48.83 with an SD of 14.90.

In the ASQ personal-social domain, children who engaged in coloring, crafting, or art activities had a substantially higher mean score of 55.75 with an SD of 6.34. Those who did not participate in these activities had a lower mean score of 46.17 with an SD of 12.23.

These results suggest that children who participated in creative and artistic activities tended to score higher on both the problem-solving and personal-social domains of the ASQ screening tool compared to those who did not engage in such activities. The difference in mean scores was more pronounced for the personal-social domain.

These findings indicate that engaging in creative and artistic activities may be associated with better problem-solving abilities and stronger personal-social skills in children.

Socio-demographics

The sample consisted of 27 males (52.9%) and 24 females (47.1%). Racial/ethnic backgrounds were 41.2% White, 29.4% Hispanic/Latino, 17.6% Black/African American, 5.9% Asian, and 5.9% other/multiracial.

## Discussion

Several studies have found that high screen time in pediatric populations has been associated with sedentary lifestyles and evidence supports an association between increased rates of obesity and increased screen time [13-16]. Additionally, several studies have found that excessive use of touchscreen technology has been associated with an increased prevalence of ophthalmologic symptoms, including dry eye disease and myopia [17-19]. Recommendations have been made regarding touchscreen technology and sleep hygiene for pediatric populations [20,15,16]. Furthermore, blue light from touchscreen devices has also been linked to precocious puberty [21].

Madigan et al. published various papers. Some of these findings include an association between screen time and poorer performance on developmental screening tests, with the hypothesis that spending more time in front of a screen takes time away from the development of communication skills [6]. In another paper, the researchers found that varying levels of screen time have been reported amongst socioeconomic populations with different amounts of exposure by the age of children [9].

Study statement

The purpose of the study is to examine associations between screen time and different screen uses on problem-solving and communication developmental skills within a low-socioeconomic population.

Though there have been studies on the effects of touchscreen usage regarding childhood milestone development, data is conflicting, making it difficult to come to a general consensus. As the use of touchscreen devices increases and becomes a necessity for so many features of our lives, this study aims to investigate the claims made between parental supervision and the positive outcomes that touchscreen technology can have on pediatric populations.

Problem statement

Little research has been conducted as to the impact of screen time on developmental stages with respect to problem-solving and communication specifically in low-income populations.

The present study investigated the relationship between touchscreen exposure and usage patterns during the critical period of early childhood development and skills measured by the ASQ-3, a widely-used developmental screening tool [11]. Specifically, we examined the timing of initial touchscreen introduction, daily duration of touchscreen use, independent touchscreen usage without adult supervision, and engagement in tactile creative activities like coloring/crafting related to performance across five key developmental domains assessed by the ASQ-3.

Our findings indicate that earlier introduction to touchscreen devices, before 12 months of age, was associated with significantly higher scores on the ASQ-3 problem-solving domain compared to later touchscreen introduction at or after 12 months. This suggests that exposure to the interactive and exploratory nature of touchscreen interfaces during the first year of life may facilitate the development of problem-solving abilities and skills like experimentation, cause-and-effect learning, and cognitive flexibility. However, we did not find significant differences in problem-solving scores based on children's average daily touchscreen usage time. The benefits of early touchscreen introduction on problem-solving development do not appear to be further enhanced or diminished by greater or lesser amounts of daily usage during the toddler years.

Our findings further revealed a notable difference in ASQ problem-solving scores based on touchscreen introduction timing. Children who were not exposed to touchscreens had the lowest mean score of 35.0 with a high SD of 20.0, indicating considerable variability in problem-solving skills within this group. In contrast, children introduced to touchscreens before 12 months of age demonstrated the highest mean score of 55.56 with a much lower SD of 9.13, suggesting more consistent and advanced problem-solving abilities. Those introduced to touchscreens at 12 months or later also showed improved performance compared to the non-exposed group, with a mean score of 51.67 and an SD of 10.29. These results further support the potential benefits of early touchscreen introduction on problem-solving development, with earlier exposure (before 12 months) appearing to confer a slight advantage over later introduction. The lower variability in scores among touchscreen-exposed children also suggests that this early interactive experience may help standardize problem-solving skill development to some extent.

For the personal-social domain of the ASQ-3, a contrasting pattern emerged. Children who were introduced to touchscreens at 12 months or later scored significantly higher on personal-social skills compared to those first exposed before 12 months of age. Additionally, moderate daily touchscreen usage between 30 and 90 minutes was associated with higher personal-social scores versus lower (<30 mins) or higher (>90 mins) usage times. These findings align with expert recommendations to limit screen exposure, including touchscreens, for infants under 12 months to avoid potential interference with the development of critical social-emotional competencies during this sensitive period. Excessive touchscreen use may impede opportunities for real-world social interactions, joint attention, and caregiver responsiveness, which are crucial for fostering personal-social skill acquisition in the first year.

Interestingly, children who usually used touchscreens independently without adult supervision had significantly higher personal-social scores compared to those who did not use touchscreens alone. Solo touchscreen play may encourage self-regulation, independence, and intrinsic motivation - skills that are reflected in the personal-social domain. Engagement in tactile creative activities like coloring, crafting, and art was also linked to better personal-social development, with children who participated in these activities scoring significantly higher. Creative play likely provides opportunities to practice cooperation, self-expression, and other social-emotional skills.

No significant differences in ASQ-3 scores were found for the communication, gross motor, or fine motor domains based on any of the touchscreen exposure or usage variables examined. This suggests that while touchscreen habits may impact problem-solving and personal-social development in early childhood, they do not appear to substantially influence the acquisition of skills in these other developmental areas during the toddler period.

A major strength of this study was the use of the well-validated and standardized ASQ-3 tool to comprehensively assess children's development across multiple domains. Additionally, detailed data on touchscreen exposure timing, daily usage patterns, independent use, and creative activity engagement were collected, allowing examination of how specific aspects of the child's experience with touchscreens related to developmental outcomes.

This study provides novel evidence that the timing, duration, context, and content of touchscreen exposure in early childhood may differentially impact specific developmental domains. While earlier touchscreen introduction before 12 months appears beneficial for problem-solving skills, later introduction at or after 12 months coupled with moderate daily usage seems optimal for fostering personal-social development. Engaging with touchscreens independently and participating in tactile creative activities were also linked to stronger personal-social competencies. These nuanced findings can help inform guidelines for touchscreen usage in early childhood and elucidate potential mechanisms by which interactive screen experiences may enrich or impede different areas of development during this critical period. Recommendations for future research include larger longitudinal studies using multi-method assessments to further clarify these complex relationships.

Several limitations should be considered when interpreting the findings of this study. First, the sample size was relatively small, consisting of only 51 parent-child dyads. This limited sample size reduces the statistical power of the study, making it more challenging to detect smaller effects that might be present. A larger and more diverse sample would not only increase the statistical power but also enhance the generalizability of the findings to a broader population.

Second, the study relied entirely on parent-report measures to gather data on children's touchscreen habits and developmental milestones. This approach is susceptible to recall biases, as parents may not accurately remember or report their children's activities. Additionally, social desirability influences could lead parents to provide responses they believe are more acceptable or favorable. To address these issues, future research should incorporate more objective measures, such as touchscreen monitoring apps that can track actual usage and direct assessments of children's developmental progress. These methods would provide a more accurate and reliable picture of the relationship between touchscreen use and child development.

Third, the cross-sectional design of the study limits the ability to make causal inferences about the impact of touchscreen exposure on development over time. Cross-sectional studies provide a snapshot of data at a single point in time, which does not allow for the examination of changes or developments that occur as a result of touchscreen use. To better understand the long-term effects of touchscreen exposure, longitudinal studies are needed. These studies would track children's touchscreen experiences and developmental trajectories over an extended period, providing insights into how early exposure might influence development in the long run.

Potential confounding factors

The study does not account for other variables that may influence child development, such as socioeconomic status or parental involvement, which could skew the results. These factors can significantly impact a child's developmental outcomes and may interact with touchscreen usage patterns in complex ways. For instance, children from higher socioeconomic backgrounds might have access to more educational touchscreen content or receive more parental guidance during screen time, potentially leading to more positive developmental outcomes regardless of screen time duration. Similarly, the level of parental involvement in a child's activities, both screen-based and non-screen-based, could be a crucial factor in determining developmental progress. Future studies should aim to control for these potential confounding variables to isolate the specific effects of touchscreen exposure on child development more accurately.

## Conclusions

This study provides novel evidence that the timing, duration, context, and content of children's touchscreen experiences in early childhood relate differently to specific areas of development. Earlier touchscreen introduction (before 12 months) was associated with higher problem-solving scores, potentially due to the interactive and exploratory nature of touchscreen interfaces facilitating skills like experimentation and understanding cause-and-effect relationships. Conversely, later introduction (at or after 12 months) was linked to stronger personal-social skills, aligning with recommendations to limit screen exposure for infants to avoid interference with crucial social-emotional learning through real-world interactions.

Notably, moderate daily touchscreen usage (30-90 minutes) and engagement in tactile creative activities were associated with better personal-social development. However, no significant relationships were found between touchscreen exposure variables and communication or motor skill domains. These nuanced findings suggest that while touchscreen habits may impact the development of problem-solving and personal-social skills in early childhood, their effects vary across developmental domains. Future research should explore potential effects at different age ranges to inform age-appropriate recommendations for supporting healthy child development in our evolving digital world.

## Appendices

Study of the Correlation and Relationship Between Early Exposure to New Technologies and the Initiation of Milestones in Early Development (SCREEN-TIMED) Questionnaire

Patient’s name: ___________________________________________________

DOB:___________________________

1. Does the patient identify as (choose as many as apply):

Black or African American

White 

Hispanic or Latino

Asian Indian

Chinese

Filipino

Japanese

Korean

Vietnamese

Native Hawaiian or Pacific Islander

Native American or Alaskan Native

Other  (please write in): ______________

2. What language do you primarily speak at home?__________________________

3. Primary caregiver’s highest level of education (choose one): 

Some high school

High school graduate

Some college

College graduate

Master’s degree

Doctoral degree

4. Does your child attend daycare, preschool or kindergarten? 

Daycare

Preschool 

Kindergarten

Homeschool (taught by parent or caregiver)

Online school (taught by teacher)

Does not attend school

Other: (comment) __________________________________________

5. For how long (in months) has your child attended preschool or kindergarten?______________

6. Do you have the following touchscreen technology in the child’s environment: tablets, smartphones, or handheld video games? If you have these devices, please specify the number of devices.

No

Yes. Number of devices ______

7. At what age (in months) was your child introduced to devices with touchscreens?___________

8. On an average day, how many minutes does your child spend holding or watching these devices: tablets, smartphones, or handheld video games? (choose one)

Less than 30 min

31-60 min

1-1 ½ hours

1 ½ -2 hours

2- 2 ½ hours

2 ½-3 hours

More than 3 hours

Not applicable (doesn’t interact with these devices)

9. On an average day, for what reason does your child use touchscreens?  (choose as many as apply)

Watching videos 

Taking selfies

Facetiming

Games 

Educational apps (e.g. learning to count, spell, read, write, or do math)

Homeschooling or online school

Other:  ________________________________________________

10. Does your child usually use their touchscreen alone or with others (choose one)?

Usually uses touchscreen alone

Usually uses touchscreen with a sibling or friend

Usually uses touchscreen with an adult 

Other: __________________________________________________

11. At what age (in months) did you first notice your child able to perform the following tasks? If they have not yet achieved, specify “not yet.” 

______ Be engaged by a touchscreen device (watch a movie, look at a photo)

______ Hold touchscreen device independently

______ Swipe right or left

______ Enlarge image

______ Shrink image

______ Unlock phone

______ Type or text

12. What activities does your child do frequently other than using a touchscreen?  (choose as many as apply)

Watching TV

Playing alone

Playing with other children or family members

Reading books or magazines

Coloring, crafting or art activities

Other _________________________________________________

13. Have you ever been told by a medical professional that your child has been diagnosed with any developmental delays or disabilities?  (choose as many as apply)

Autism or Autism Spectrum Disorders

ADHD (Attention-Deficit/Hyperactivity Disorder)

Speech or Language delay

Fine motor delay

Gross motor delay

Cognitive impairment/Intellectual disability

14. Was your child born prematurely? (choose one)

Full Term

Premature (35-37 weeks)

Premature (30-34 weeks)

Premature (25-29 weeks)

Premature (before 24 weeks)

15. Is your child receiving any therapies?  (choose as many as apply)

Physical therapy

Occupational therapy

Speech therapy

ABA (Applied Behavioral Analysis) therapy

Other:  __________________________________________
